# Adverse outcome associated with daratumumab-based treatments in relapsed/refractory multiple myeloma patients with amplification of chromosome arm 1q21: a single-center retrospective experience

**DOI:** 10.1007/s00277-022-04978-6

**Published:** 2022-09-15

**Authors:** Emiliano Barbieri, Monica Maccaferri, Giovanna Leonardi, Francesca Giacobbi, Giorgia Corradini, Ivana Lagreca, Patrizia Barozzi, Leonardo Potenza, Roberto Marasca, Mario Luppi

**Affiliations:** Hematology Unit and Chair, Azienda Ospedaliera Universitaria Di Modena and Department of Medical and Surgical Sciences, University of Modena and Reggio Emilia, Modena, Italy

**Keywords:** Chromosome 1q gain/amplification, Chromosome 1q amplification, Multiple myeloma, Relapsed-refractory multiple myeloma, Daratumumab, To the editor

Gain/amplification of chromosome arm 1q21 (1q21 +) is among the most common abnormalities found in multiple myeloma (MM) and is by now almost universally recognized as a poor prognostic factor in MM patients, recently included among the top features of the second revision of the International Staging System (R2-ISS) [1, 2]. 1q21 + can be divided into gain (gain1q) or amplification of 1q21 (amp1q), respectively, if three or more than three copies of 1q21 are detected, and the distinction between these two categories has increasingly become more important since the copy number of 1q21 seems to reflect the genomic instability, proliferation rate, drug resistance, and early progression/death rate of the disease [1, 3, 4]. Nevertheless, the optimal management of 1q21 + patients in the era of novel therapies has yet to be determined [1, 5, 6]. Daratumumab, an anti-CD38 monoclonal antibody, has provided clear demonstration of efficacy in MM patients, even among high-risk (HR) MM patients, and is now licensed for use in relapsed-refractory MM (RRMM) as well as newly diagnosed MM (NDMM) [7, 8]. However, data supporting the effectiveness of adjunctive daratumumab in 1q21 + patients are scarce, since pivotal trials didn’t report outcomes on this patient population, and potential mechanisms of resistance conferred by 1q21 + to daratumumab therapy have been proposed [1]. Interestingly, Hu and colleagues [9] have recently published a retrospective study describing real-world outcomes of 34 NDMM patients harboring 1q21 + , reporting no prognostic role of daratumumab therapy and worse outcomes for amp1q patients compared to gain1q patients. As the authors noted, only one study by Mohan and colleagues [10] had previously investigated the outcomes of 1q21 + patients treated with daratumumab, reporting poor prognosis associated with both 1q21 + and GEP-70 HR status, although patients in that study were heavily pretreated and only 63% of them received daratumumab in combinations with immunomodulatory drugs.

In this letter, we report a retrospective series of 8 consecutive RRMM patients harboring amp1q who received treatment at our institution with daratumumab-based triplet regimens at first (*n* = 7) or second (*n* = 1) relapse, with very dismal outcomes (Table S1). Interphase FISH was performed using locus-specific DNA probe 1q21/8p21 dual color FISH (Empire Genomics). The study was approved by local Ethical Committee (Protocol 0,036,084/21). On the data cutoff date 30 November 2021, after a median follow-up of 10.1 months (3.0–17.7), median PFS was 3.0 months (1.6–7.6), only 1 patient achieved very good partial response (VGPR), 7 patients discontinued treatment, all due to disease progression, and 4 have died. By contrast, during the same period, for RRMM patients without amp1q (*n* = 40) who received daratumumab-based rescues at comparable disease stages, the median PFS was not reached after a median follow-up of 18.4 months (2.3–39.4) (Fig. 1) (Table S2). Our findings support data on poor outcomes associated with daratumumab treatment in amp1q patients, even when daratumumab-based regimens are used very early in the treatment course. We believe that these patients should be considered for novel treatment strategies.

**Fig. 1:**
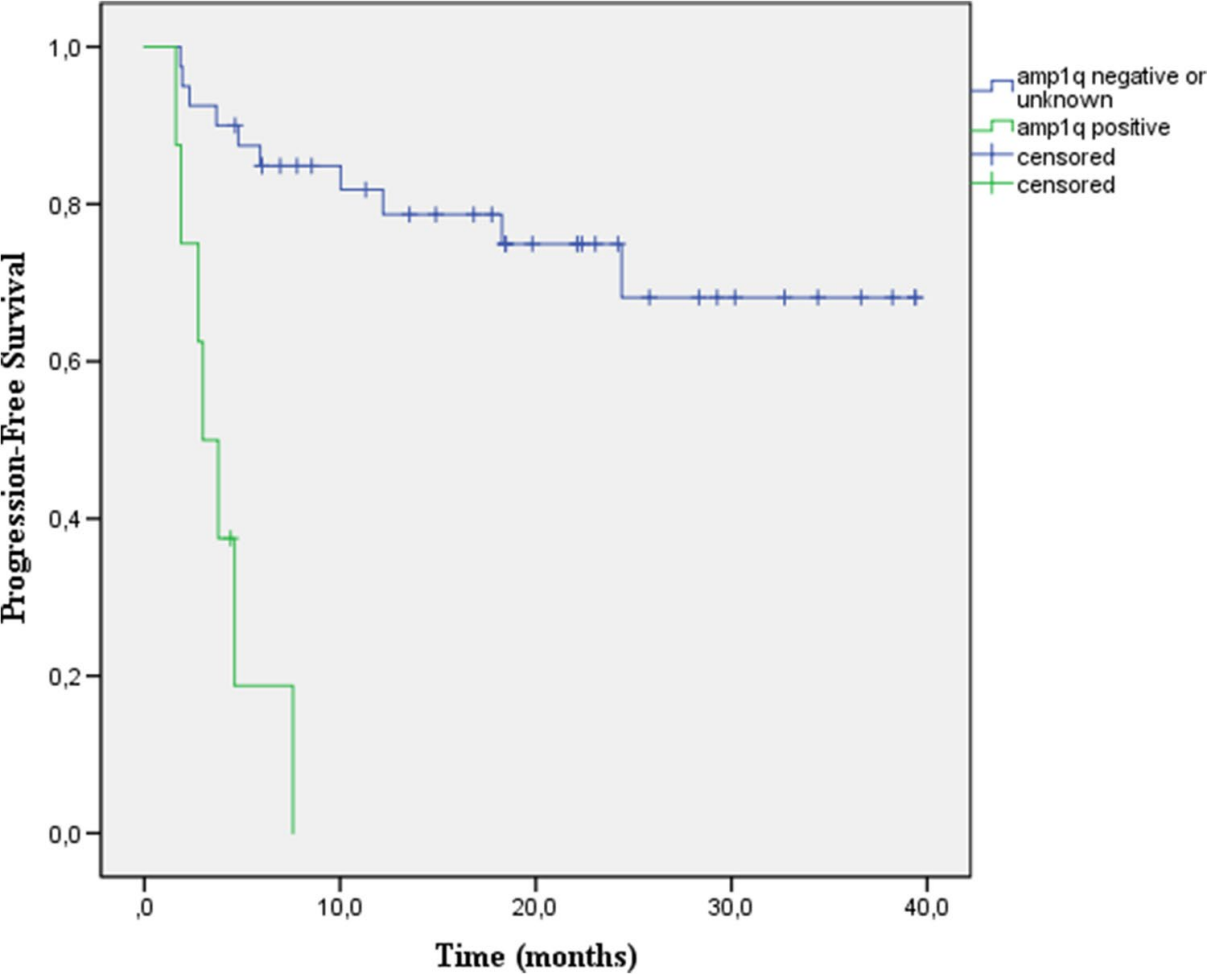
Kaplan–Meier curves showing progression-free survival in the groups stratified by amplification of chromosome arm 1q21

## Supplementary Information

Below is the link to the electronic supplementary material.

Supplementary file1 (DOCX 22 KB)

## Data Availability

The data that support the findings of this study are available from the corresponding author [EB], upon reasonable request.

## References

[1] Bisht K, Walker B, Kumar SK (2021). Chromosomal 1q21 abnormalities in multiple myeloma: a review of translational, clinical research, and therapeutic strategies. Expert Rev Hematol.

[2] D’Agostino M, Cairns DA, Lahuerta JJ (2022). Second revision of the international staging system (R2-ISS) for overall survival in multiple myeloma A European myeloma network (EMN) report within the HARMONY project. J Clin Oncol.

[3] Hanamura I (2021) Gain/amplification of chromosome arm 1q21 in multiple myeloma. Cancers 13. 10.3390/cancers13020256 10.3390/cancers13020256 PMC7827173 33445467

[4] Schmidt TM, Fonseca R, Usmani SZ (2021). Chromosome 1q21 abnormalities in multiple myeloma. Blood Cancer J.

[5] Dimopoulos MA, Moreau P, Terpos E (2021). Multiple myeloma: EHA-ESMO clinical practice guidelines for diagnosis, treatment and follow-up. Ann Oncol.

[6] Rajkumar SV (2022). Multiple myeloma: 2022 update on diagnosis, risk stratification, and management. Am J Hematol.

[7] Premkumar V, Pan S, Lentzsch S, Bhutani D (2020) Use of daratumumab in high risk multiple myeloma A meta-analysis. eJHaem 1:267–271. 10.1002/jha2.47 10.1002/jha2.47 PMC9175911 35847747

[8] Giri S, Grimshaw A, Bal S (2020). Evaluation of daratumumab for the treatment of multiple myeloma in patients with high-risk cytogenetic factors. JAMA Oncol.

[9] Hu X, Wu C-H, Cowan JM (2022). Outcomes of patients with multiple myeloma harboring chromosome 1q gain/amplification in the era of modern therapy. Ann Hematol.

[10] Mohan M, Weinhold N, Schinke C (2020). Daratumumab in high-risk relapsed/refractory multiple myeloma patients: adverse effect of chromosome 1q21 gain/amplification and GEP70 status on outcome. Br J Haematol.

