# Supplementary material for: Adverse outcome associated with daratumumab-based treatments in relapsed/refractory multiple myeloma patients with amplification of chromosome arm 1q21: a single-center retrospective experience
Source: Ann Hematol. 2022 Sep 15;101(12):2777–9. doi: 10.1007/s00277-022-04978-6 (PMC9646578; doi:10.1007/s00277-022-04978-6)
Supplement: Supplementary file 1 — Supplementary file1 (DOCX 22 KB) [file 277_2022_4978_MOESM1_ESM.docx]

SUPPLEMENTAL MATERIAL

**Table S1.** Disease characteristics and outcomes of amp1q patients treated with daratumumab-based rescues as second- or third-line therapy at our center.

**Table S2**. Disease characteristics and outcomes of patients without amp(1q) who received second- or third-line daratumumab-based salvage regimens at our center.

| MM= multiple myeloma. CA= cytogenetic abnormality. Amp1q= amplification of chromosome arm 1q21. DBT= daratumumab-based triplet. N= number. PFS= progression-free survival. TNT= time to next treatment. N/A= not available. DRD= daratumumab/lenalidomide/dexamethasone. DVD= daratumumab/bortezomib/dexamethasone. PR= partial response. VGPR= very good partial response. PD= progressive disease. MinR= minimal response. N/A= not applicable. VTD= bortezomib/thalidomide/dexamethasone. ASCT= autologous stem cell transplant. MR= maintenance lenalidomide. KD= carfilzomib/dexamethasone. VCD= bortezomib/cyclophosphamide/dexamethasone. KCD= carfilzomib/cyclophosphamide/dexamethasone. VMP= bortzemobi/melphalan/prednisone. PVD= pomalidomide/bortezomib/dexamethasone. BM= belantamab mafodotin. PAD= bortezomib/dexamethasone/doxorubicin. PCD= pomalidomide/cyclophosphamide/dexamethasone. PACE= cisplatin/doxorubicin/cyclophosphamide/etoposide. ^a^ patients with high-risk CAs had at least one of the following high-risk CAs [del(17p), t(14;16), or t(4;14)]. ^b^ VTD induction treatment was switched to VCD after one cycle due to patient intolerance to thalidomide. ^c^ VTD induction treatment was switched to VMP after two cycles due to patient intolerance to thalidomide. | Median (range) | Patient 8 | Patient 7 | Patient 6 | Patient 5 | Patient 4 | Patient 3 | Patient 2 | Patient 1 | Patient | Table S1. Disease characteristics and outcomes of amp1q patients treated with daratumumab-based rescues as second- or third-line therapy at our center. |
| --- | --- | --- | --- | --- | --- | --- | --- | --- | --- | --- | --- |
|  | 65.5 (51 - 72) | 66 | 67 | 72 | 51 | 56 | 53 | 65 | 70 | Age - years |  |
|  |  | Female | Male | Male | Female | Male | Male | Male | Male | Sex |  |
|  |  | IgG kappa | IgA lambda | IgG lambda | IgA lambda | Light-chain | IgA lambda | Light-chain | IgA lambda | Type of MM |  |
|  |  | del(17p) | No | No | No | No | t(4;14) | No | No | High-risk CAs^a^ |  |
|  |  | DRD | DRD | DRD | DVD | DRD | DRD | DRD | DRD | DBT rescue regimen |  |
|  | 13.5 (6 - 25) | 9 | 13 | 25 | 6 | 17 | 14 | 17 | 12 | No. of daratumumab infusion |  |
|  | 5.5 (1 - 10) | 2 | 6 | 10 | 1 | 6 | 5 | 6 | 3 | No. of cycles completed |  |
|  |  | PD | PR | MinR | PD | VGPR | PR | PR | PR | Best response |  |
|  | 3.0 (1.6 -7.6) | 1.9 | DBT therapy ongoing | 7.6 | 1.6 | 2.8 | 3.8 | 4.6 | 3.0 | PFS - months |  |
|  | 4.0 (1.7 - 6.5) | 2.5 | DBT therapy ongoing | N/A | 1.7 | 5.8 | 4.6 | 6.5 | 3.4 | TNT - months |  |
|  |  | 1 | 1 | 1 | 1 | 1 | 1 | 1 | 2 | No. of previous lines of therapy |  |
|  |  | 1^st^ line: VTD + ASCT + MR | 1^st^ line: VTD + ASCT | 1^st^ line: VTD/VMP^c^ | 1^st^ line: VTD + 2ASCT + MR | 1^st^ line: VTD + 2ASCT | 1^st^ line: KCD + ASCT + KCD + MR | 1^st^ line: VTD/VCD^b^ | 1^st^ line: VTD + ASCT + MR  2^nd^ line: KD | Previous lines of therapy |  |
|  | 40.4 (9.4 - 71.4) | 45.1 | 66.5 | 71.4 | 35.6 | 49.0 | 27.3 | 9.4 | 27.2 | Time from first-line therapy to DBT rescue - months |  |
|  | 2 (0 - 4) | 1 | N/A | 0 | 2 | 1 | 4 | 3 | 2 | No. of subsequent lines |  |
|  |  | 3^rd^ line: KD | N/A | N/A | 3^rd^ line: KRD; 4^th^ line: PCD | 3^rd^ line: PVD | 3^rd^ line: KD;  4^th^ line: PACE;  5^th^ line: CAR-T;  6^th^ line: PVD | 3^rd^ line: KD;  4^th^ line: PAD;  5^th^ line: PCD | 4^th^ line: PVD;  5^th^ line: BM | Subsequent lines of therapy |  |
|  |  | alive | alive | 14.1 | alive | 7.6 | 17.7 | alive | 12.5 | OS - months |  |
|  | 10.1 (3.0 - 17.7) | 3.0 | 4.4 | 14.1 | 7.2 | 7.6 | 17.7 | 17.5 | 12.5 | Follow-up - months |  |

| Table S2. Disease characteristics and outcomes of patients without amp1q who received second- or third-line daratumumab-based salvage regimens at our center (n = 40) | | |
| --- | --- | --- |
| Characteristic | | No. of patients or median (Range) |
| Age | | 63.5 (49 – 80) |
| Line of therapy | 2  3 | 30  10 |
| Therapy associated with daratumumab | Lenalidomide  Bortezomib | 34  6 |
| Patients still receiving DBT therapy | Yes  No | 28  12 |
| If not, reason for discontinuation | Progression  Death | 7  2  3 |
|  | Other |  |
| Patients discontinuing DBT treatment | Median no. of subsequent lines of therapy  Median TNT | 1 (0 – 2)  7.4 (2.3 – 20.7) |
| Median time from first-line treatment to DBT treatment |  | 48.0 (5.3 – 172.9) |
| Previous exposure to PIs | Yes  Bortezomib  Carfilzomib  Both No | 37  36  7  6  3 |
| Previous exposure to IMIDs | Yes  Thalidomide  Lenalidomide  Both  No | 28  24  8  4  12 |
| Previous ASCT | 0  1  2 | 13  11  15 |
| Cytogenetic risk | Standard  High^a^  Unknown | 20  1  19 |
| Patient status | Alive  Dead | 36  4 |
| Median PFS |  | Not reached |
| Median Follow-Up |  | 18.4 (2.3 – 39.4) |
| Amp1q= amplification of chromosome arm 1q21. DBT = daratumumab-based triplet. TNT = time to next treatment. PI = proteasome inhibitor. IMID = immunomodulatory drug. ASCT = autologous stem cell transplant. PFS = progression-free survival. ^a^ Cytogenetic risk was based on fluorescence in situ hybridization and/or karyotype analysis; patients were considered to have a high-risk cytogenetic profile if they had at least one of the following high-risk abnormalities: del(17p), t(14;16), or t(4;14). | | |
